# Leveraging Genomics, Transcriptomics, and Epigenomics to Understand the Biology and Chemoresistance of Ovarian Cancer

**DOI:** 10.3390/cancers13164029

**Published:** 2021-08-10

**Authors:** Sandra Muñoz-Galván, Amancio Carnero

**Affiliations:** 1 Instituto de Biomedicina de Sevilla, IBIS, Hospital Universitario Virgen del Rocío, Universidad de Sevilla, Consejo Superior de Investigaciones Científicas, Avda. Manuel Siurot s/n, 41013 Seville, Spain; 2 CIBERONC, Instituto de Salud Carlos III, 28029 Madrid, Spain

**Keywords:** ovarian cancer, genomics, transcriptomic, epigenomics, chemoresistance

## Abstract

**Simple Summary:**

Ovarian cancer is the leading cause of death by a gynecological tumor, mainly due to its common detection in advanced stages and to its high resistance to conventional chemotherapy. The study of the molecular mechanisms leading to ovarian tumor progression and chemoresistance are thus a priority in oncological research. In recent years, omics technologies have allowed the study of new aspects of ovarian cancer biology from a global point of view. In this review, we aim to summarize the main findings reached by recent studies in ovarian cancer using genomic, transcriptomic, and epigenomic techniques and how they have improved our understanding of ovarian cancer biology and chemoresistance.

**Abstract:**

Ovarian cancer is a major cause of fatality due to a gynecological malignancy. This lethality is largely due to the unspecific clinical manifestations of ovarian cancer, which lead to late detection and to high resistance to conventional therapies based on platinum. In recent years, we have advanced our understanding of the mechanisms provoking tumor relapse, and the advent of so-called omics technologies has provided exceptional tools to evaluate molecular mechanisms leading to therapy resistance in ovarian cancer. Here, we review the contribution of genomics, transcriptomics, and epigenomics techniques to our knowledge about the biology and molecular features of ovarian cancers, with a focus on therapy resistance. The use of these technologies to identify molecular markers and mechanisms leading to chemoresistance in these tumors is discussed, as well as potential further applications.

## 1. Introduction

Ovarian cancer (OC) is a gynecological malignancy responsible for 4.4% of cancer-related deaths in the world and is the most lethal gynecological tumor [1]. The five-year overall survival of epithelial OC patients ranges from 20% at stage IV to 89% at stage I, but 80% of patients are diagnosed at advanced stages (III or IV) due to unspecific clinical manifestations [2]. The standard treatment for OC consists of cytoreductive surgery combined with chemotherapy based on platinum (cisplatin or carboplatin) alone or in combination with paclitaxel, doxorubicin, or docetaxel, as well as poly (ADP-ribose) polymerase (PARP) inhibitors for patients with mutations in BRCA1 or BRCA2 [3]. Adjuvant intraperitoneal chemotherapy is also part of the standard treatment due to the poor drug distribution to the intraperitoneal cavity [4]. Histologically, there are three major OC types: epithelial tumors, which comprises 90% of cases; germ cell tumors, comprising 3% of cases; and sex cord-stromal tumors, comprising 2% of cases; with the remaining 5% of tumors’ type not specified (Table 1) [2]. Epithelial OCs comprise four subtypes: serous, which could be high-grade serous (HG-SOC) or low-grade serous (LG-SOC); endometroid; clear cell; and mucinous [5]. While type I tumors, comprising LG-SOC, endometroid, clear cell, and mucinous carcinoma, as well as nonepithelial tumors, show less malignancy and genome instability, type II tumors comprise HG-SOC and are highly malignant and genetically unstable (Table 1) [6]. HG-SOC constitutes 75% of total OC cases and therefore has been extensively studied.

**Figure 1:**
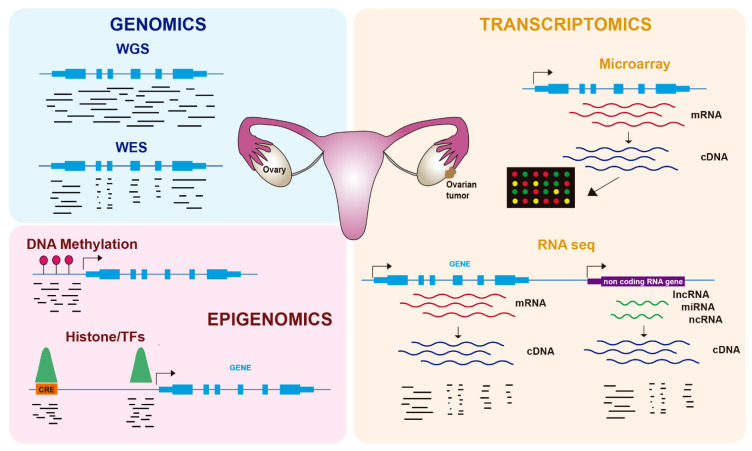
Genomic, transcriptomic, and epigenomic techniques have revolutionized our understanding of OC. The main approaches to inspect the genomic landscape of OC are whole-genome sequencing (WGS), which consists of sequencing of both coding and noncoding DNA, and whole-exome sequencing (WES), in which exons are captured and sequenced at high coverage. Transcriptomic approaches include microarray platforms, which quantify mRNA expression levels using fluorescent probes, and RNA-seq, which sequences total cDNA from both mRNAs and ncRNAs. Epigenomic approaches comprise the study of global DNA methylation, using techniques such as bisulfite sequencing, and the genome-wide enrichment of histone modifications or TF binding by ChIP-seq.

In the last twenty years, genome-wide technologies have considerably advanced our knowledge about the molecular features of human cancers, including OC. In particular, the development of next-generation sequencing (NGS) has revolutionized how we investigate many biological questions. The ability to inspect whole genomes, transcriptomes, or different aspects of the epigenome in a single experiment has allowed an unprecedented increase in our understanding of how tumorigenesis works and the mechanisms by which cancer cells proliferate and avoid cellular controls and anticancer treatments. In this review, we cover recent advances in the molecular biology of OC using genomics, transcriptomics, and epigenomics (Figure 1), with a focus on its biological features and therapy resistance.

## 2. Genomics of OC

Genomic studies in OC have used two major approaches: whole-exome sequencing (WES) and whole-genome sequencing (WGS). WES involves the capture of DNA coding regions and their in-depth sequencing and has been used to detect disease-causing genetic variants and novel target genes [7]. Exome capture allows the sequencing of coding regions at deep coverage (Figure 1), and for that reason, WES is the most commonly used NGS technique for the analysis of the genomic landscape associated with disease. However, WES has the limitation of being restricted to exons and therefore misses alterations in noncoding regions, such as regulatory elements, that have been involved in cancer [8]. In contrast, WGS provides the most complete analysis of the genomic landscape by sequencing the entire coding and noncoding genome (Figure 1). The major limitations of WGS are the requirement of more starting material and the increase in sequencing costs, as well as the use of more complex bioinformatic tools to analyze the vast amount of generated data. However, its higher power has allowed the identification of rare disease-associated variants [9]. Targeted gene panels, which consist of the capture of a series of candidate genes, have been a cheaper and cost-effective alternative to WES and WGS, although they are unable to detect new target genes beyond the captured genes [10].

### 2.1. OC Genomic Alterations: Mutations, Copy Number Variations and Structural Variants

Several studies have used WES or WGS to identify genomic alterations involved in OC tumorigenesis, including somatic and germline mutations, copy number alterations (CNAs), and structural variants (SVs). The Cancer Genome Atlas (TCGA) study involving HG-SOC analyzed 489 patients’ mRNA and miRNA expression, promoter DNA methylation, and copy number, as well as WES in 316 of them and matched them with normal DNA samples [11]. They found TP53 mutations in virtually all tumors (96%), consistent with previous data [12], but other mutations were infrequent in HG-SOC. BRCA1 and BRCA2 showed germline mutations in 8 and 9% of cases, respectively, and somatic mutations in 3% of cases. Six additional genes showed statistically significant recurrent mutations in 2–6% of cases: RB1, NF1, FAT3, CSMD3, GABRA6, and CDK12. Finally, they described other rare mutations involving important HG-SOC drivers, such as BRAF, PIK3CA, KRAS, and NRAS. Furthermore, 113 CNAs were identified, including focal amplifications of CCNE1, MYC, MECOM, ZMYND8, IRF2BP2, ID4, PAX8, and TERT, as well as focal deletions of tumor suppressor genes such as PTEN, RB1, and NF1, the latter of which were also significantly mutated [11]. Integrative pathway analyses combining expression, methylation, and mutation showed altered pathways in HG-SOC, including RB1, PIK3/RAS, NOTCH, and FOXM1, and homologous recombination (HR) defects were also found for half of the analyzed tumors.

Subsequent studies in HG-SOC confirmed the main results of the TCGA study and identified new alterations. In this regard, Patch et al. used WGS together with transcriptome and methylome analyses in 92 patients [13]. They confirmed the prevalence of TP53 mutations in HG-SOC, as well as mutations in HR genes or BRCA1 promoter methylation in half of the tumors. They also identified gene breakage in tumor suppressor genes such as RB1, NF1, RAD51B, and PTEN, as well as CCNE1 amplification as the primary SV, consistent with previous results [14]. In addition to primary disease, this study also analyzed metastases to ascites for tumors sensitive to initial treatment but failing subsequent therapy. No clear relationship with chemoresistance was evident for the genes specifically mutated in the ascites tumoral cells; however, particular patients showed either reversions of BRCA1-2 mutations, similar to previous studies [15,16], loss of BRCA1 promoter methylation, or promoter fusions overexpressing the ABCB1 gene encoding the drug efflux transporter MDR1, which mediates rapid efflux of chemotherapeutic agents, including paclitaxel [17]. A larger study used WES with 2051 OC patients and found four susceptibility genes for OC: MSH6, RAD51C, TP53, and ATM [18], confirming the alterations in DNA repair found in OC.

Shallow whole-genome DNA sequencing was applied to 117 samples to study copy number signatures of HG-SOC [19]. This report described six copy number signatures underlying mutational processes in ovarian tumors occurring in parallel after widespread TP53 mutation. These signatures were associated with particular driver mutations and disease outcomes, including overall survival and tumor relapse after platinum treatment. For instance, copy number signature 3 is characterized by BRCA1-2-related HR defects and improved overall survival, consistent with previous data [20,21], while copy number signature 1 is characterized by oncogenic RAS signaling (mutations in NF1, KRAS, and NRAS) and predicts platinum-resistant relapse and poor survival.

### 2.2. OC Tumor Heterogenety and Evolution

Genomic techniques have also been used for studies on tumor evolution and heterogeneity. Several reports have analyzed the genomic landscape of OC metastases and reached the common conclusion that there is little accumulation of somatic mutations and CNAs in metastatic versus primary tumor samples. Lee et al. used 11 samples from primary and metastatic sites in a single HG-SOC patient [22]. By performing WES and sample clustering, they obtained three clusters: one of metastatic samples and two of primary tumor samples, where the metastatic samples arose from one of the primary tumor clusters with few additional somatic mutations and CNAs. Moreover, Schwarz et al. quantified the degree of tumor heterogeneity in 14 HG-SOC patients using WGS [23]. They found that a high degree of clonal expansion was associated with worse survival and that clonal populations detected at relapse originated in early branching events followed by divergent clonal evolution, which is consistent with previous observations showing that chemotherapy did not lead to substantial changes in genetic subclones and that genetically divergent subclones resistant to platinum may exist in the tumor before treatment [24].

Another study explored the modes of clonal spread by HG-SOC at peritoneal metastases [25]. They analyzed the proportion of local spread in the peritoneum arising from monoclonal seeding and expansion or from extensive cellular mixing of diverse polyclonal populations. For that analysis, they used WGS and single-nucleus sequencing in 68 samples from seven patients, including samples from the primary sites as well as metastases to the omentum, fallopian tubes, peritoneum, and other distal sites. They found at least two different modes of tumor spreading to metastatic sites: extensive migration and polyclonal reseeding at multiple sites, which was observed in two out of seven patients, and monoclonal and unidirectional seeding from the ovary to metastatic sites, which was observed in five out of seven patients, challenging the selection capacity attributed to the microenvironment of the peritoneal cavity.

The immune microenvironment of ovarian tumors was analyzed in relation to patient outcome by other studies. Zhang et al. performed a high-resolution multi-site profiling of immune and malignant populations in the HG-SOC tumor microenvironment [26]. They analyzed tumor-infiltrating lymphocytes in 212 samples from 38 HG-SOC patients and found a high intra-patient variation in the immune responses across different intra-peritoneal sites. Moreover, they found that high levels of epithelial immune infiltration were associated with low malignant clone diversity, suggesting a purifying selection process, and that the combination of immune activity and mutational processes predicted patient prognosis, with high immune activity and low foldback inversions being associated with better outcomes. Another study focused on HR proficient HG-SOC tumors, which do not benefit from therapy with PARP inhibitors. They found a group of tumors with high immunological activity (high neoantigen load and high HLA class I expression) that showed a better prognosis and proposed therapy with immune checkpoint inhibitors for them [27].

The ovarian surface epithelium may lead to OC in a minority of cases, but most epithelial OCs originate from neoplastic lesions at the Fallopian tube epithelium (FTE) [28,29]. Indeed, driver mutations have been detected both in tumors and in FTE. These early lesions, called serous tubal intraepithelial carcinomas (STICs), show features similar to the main lesion, such as TP53 mutations and upregulation of cell cycle and DNA repair genes [30,31]. In this sense, Hellner et al. collected micrometastases and microscopic residual disease samples from an HG-SOC patient for three years to identify early driver mutations [32]. They found frequent mutations in a region containing repressors of the SOX2 stem marker gene, and this region was already mutated in the FTE of the patient, being almost ubiquitous in HG-SOC patients. They propose SOX2 expression in FTE as an early marker to detect disease at premalignant stages. In contrast, another study analyzed the genomic landscape of HG-SOC and STICs in four patients by WES and amplicon sequencing [33] and found nonidentical TP53 mutations between STICs and tumors within the same individuals, supporting a model of multiple clonal origins of tubal lesions at early stages of tumorigenesis. The proliferation rates suggested longer times for developing the p53 signature and STICs (approximately two decades) than for STIC progression to carcinoma (approximately six years).

### 2.3. OC Genomics and Chemoresistance

Several studies have used genomic approaches to search for genetic determinants of chemoresistance in OC with heterogeneous results (Table 2). In HG-SOC, the study by Patch et al. (see above) found only some associations with therapy resistance in particular patients [13]. These associations with therapy resistance included reversions of BRCA1-2 mutations, loss of BRCA1 promoter methylation, and promoter fusions resulting in the overexpression of the drug efflux transporter MDR1. Interestingly, a patient with five independent reversions of BRCA2 germline mutations was non-responsive to the PARP inhibitor olaparib and carboplatin. Another study using patient data from TCGA found mutations in ADAMTS superfamily genes in 10.4% of cases [34]. These mutations were associated with better survival and higher platinum sensitivity. Recently, a genomic signature was reported to predict platinum resistance in HG-SOC patients [35]. By WGS and WES, they identified mutations in TP53 and TTN, as well as defects in HR repair, as the most common genomic alterations in both platinum-sensitive and platinum-resistant patients. A predictor based on 14 genomic parameters, such as copy number, SNV load, duplication load, or homologous recombination defects, called DRDscore, was used to determine platinum sensitivity. This score was defined in a discovery cohort of 57 patients and validated in another 42 patients with 90.9% accuracy.

Apart from platinum-based therapy, other studies have focused on the genomics of chemoresistance to alternative treatments, such as PARP inhibitors. The analysis of WES from 220 TCGA samples without BRCA mutations identified RAD50 deletions in 18% of samples, showing significantly better survival [36]. The knockdown of RAD50 in OC cell lines led to sensitivity to the PARP inhibitors olaparib and rucaparib, suggesting RAD50 as a candidate marker for PARP inhibitor response in OC patients. Another study identified OC cell lines carrying MYC amplifications as more sensitive to the PARP inhibitor BMN673 [37]. More recently, a method to detect a gene signature of homologous recombination (HR) deficiency, which uses targeted gene panels instead of WES or WGS, was developed [38]. Cell lines identified as HR-deficient with this method showed sensitivity to PARP inhibitors, and OC patients with HR deficiency showed a longer survival after platinum treatments.

Other studies have analyzed the chemoresistance to paclitaxel alone or in combination with platinum. Li et al. established chemosensitive and chemoresistant PDX models and investigated gene expression and mutations associated with chemoresistance to paclitaxel and carboplatin [39]. They identified the differential expression of SAP25, HLA-DPA1, AKT3, and PIK3R5 and the mutation of TMEM205 and POLR2A as important for the progression of chemoresistance. Recently, a study analyzed genomic aberrations associated with the response to weekly paclitaxel by shallow WGS [40]. They found no differences between short and long responders. Only in patients with exceptionally long responses were amplification of angiogenesis, tubulin, and interleukin genes detected.

Studies using other chemotherapies include combined treatment with platinum and antiHER2 therapy, which obtained better results than both therapies alone in PDX models of HG-SOC with alterations in genes encoding members of the ERBB2 pathway [41]. In addition, a study used WES of LG-SOC patient-derived cell lines to analyze the effect of MEK inhibitors. MEKi-sensitive cell lines are KRAS mutants, and MEKi-resistant cell lines express high levels of EGFR and PKC-alpha proteins [42]. Combined treatment with MEKi and EGFRi in the resistant lines showed better results. Finally, a patient with recurrent HG-SOC resistant to chemotherapy and radiotherapy was analyzed by a recent report, showing an extraordinary response to the PD1 immune checkpoint inhibitor pembrolizumab [43]. This patient presented a low number of mutations but carried a structural variant disrupting the 3′ region of the PD-L1 gene that caused its aberrant surface expression, suggesting immune evasion as the most likely mechanism for therapy resistance.

**Table 2:** Markers of OC chemoresistance identified by genomic, transcriptomic, and epigenomic techniques.

Marker	Approach	Alteration	Treatment	Outcome	OC Subtype	Refs.
*BRCA1, BRCA2*	WGS	Mutation reversion	Platinum	Resistance	HG-SOC	[13]
ADAMTS Family	WES	Mutation	Platinum	Sensitivity	HG-SOC	[34]
*TMEM205,* *POLR2A*	WES	Mutation	Paclitaxel + Carboplatin	Resistance	HG-SOC	[39]
*PDL-L1*	WES	Structural variant	Pembrolizumab	Sensitivity	HG-SOC	[43]
*KRAS*	WES, WGS	Mutation	MEK inhibitors	Sensitivity	LG-SOC	[42]
*MDR1*	RNA-seq	Up-regulated expression	Platinum	Resistance	HG-SOC	[13]
*SAP25* *HLA-DPA1* *AKT3* *PIK3R5*	RNA-seq	Differential expression	Paclitaxel + Carboplatin	Resistance	HG-SOC	[39]
*IRF1*	RNA-seq	Up-regulated expression	Platinum	Sensitivity	HG-SOC	[44]
*DUOXA1*	RNA-seq	Up-regulated expression	Platinum	Resistance	Ovarian cell line	[45]
*miR-137*	RNA-seq	Down-regulated expression	Cisplatin	Resistance	Ovarian cell line	[46]
*HIF1A-AS2*	Bru-seq	Up-regulated expression	Olaparid + carboplatin + cisplatin	Sensitivity	Ovarian cell line	[47]
*BRCA1*	Bisulfite chip	Loss of promoter methylation	Platinum	Resistance	HG-SOC	[13]
H3K27me3/H3K4me3	ChIP-seq, RNA-seq	Down-regulated expression	Platinum	Resistance	HG-SOC	[48]
H3K79me	ChIP-seq	Increased deposition	Platinum	Resistance	Ovarian cell line	[49]
*SOX9*	ChIP-seq RNA-seq	Up-regulated expression by superenhancers	Cisplatin	Resistance	Ovarian cell line	[50]
*ISL1*	ChIP-seq RNA-seq	Down-regulated expression by superenhancers	Cisplatin	resistance	Ovarian cell line	[51]

## 3. Transcriptomics of OC

Gene expression was initially characterized in OC and other tumors using microarray-based technologies (Figure 1), leading to the identification of several molecular subtypes. Tothill et al. profiled 285 serous and endometroid ovarian tumors and identified six clusters of ovarian tumors with distinct molecular features and malignant potential: two clusters of low-grade and four clusters of high-grade tumors [52]. These tumors included a novel high-grade serous subtype characterized by a mesenchymal cell state. In the TCGA study, 11,864 genes were analyzed by three different microarray-based platforms, leading to 1500 variable genes that were used to obtain four clusters: immunoreactive, differentiated, proliferative, and mesenchymal tumors [11]. Importantly, they identified a transcriptional signature formed by 193 genes, 108 of which were correlated with poor patient survival and 85 with good survival.

More recently, the development of NGS technologies has revolutionized transcriptomic analyses. RNA sequencing (RNA-seq) allows the quantification of total RNA molecules in cells, thus overcoming the limitations of microarray-based technologies to a limited set of gene probes and expanding to the detection of noncoding RNAs (ncRNAs), including microRNAs (miRNAs), long noncoding RNAs (lncRNAs), and other types [53] (Figure 1). In the next subsections, we will summarize the contribution of RNA-seq to our knowledge of the origin of OC, biomarkers for early detection, and therapy resistance in OC.

### 3.1. Biomarkers for Detection and Outcome of OC

The origin of ovarian tumors in FTE [28] has been supported by studies based on RNA-seq. Qiu et al. analyzed gene expression in 31 tissue samples, including HG-SOC, LG-SOC, FTE, ovarian surface epithelium, and peritoneal mesothelia. They found that LG-SOC and HG-SOC samples clustered together with FTE and showed consistent overexpression of markers such as PAX8, CDH1, and FOXA2, suggesting a clonal origin of both HG-SOC and LG-SOC in FTE [54]. Indeed, a study using 102 human cancer cell lines previously identified PAX8 as differentially expressed in OC cell lines [55]. PAX8 is amplified in 16% of HG-SOC patients and overexpressed in ovarian tumors, suggesting that PAX8 is a lineage-specific essential gene, at least in a subset of OC cases. More recently, the transcription factor SOX18 has also been involved in the development of ovarian tumors from FTE [56].

The only OC biomarker currently approved for clinical use in early detection is circulating cancer antigen 125 (CA125), although it is common to other tumors, and it is only overexpressed in a subset of early stage tumors [57]. Therefore, there is a necessity to identify more specific and early expressed biomarkers of OC detection. In this regard, insulin-like growth factor binding protein 4 (IGFBP-4) was identified as upregulated in serous OC samples using RNA-seq, including early stage patients in which CA125 levels were normal [58]. Moreover, IGFBP-4 levels were higher in malignant than in benign disease. Another study analyzed the involvement of the solute carrier (SLC) family in OC patients, identifying SLC7A2 as downregulated and associated with poor prognosis [59]. Finally, Fei et al. analyzed published OC patient data and found that KIF11, CDC20, and TOPA were upregulated in ovarian tumors and correlated with worse patient survival [60].

Apart from mRNA, RNA-seq technology has the advantage of detecting ncRNAs at the same time, and some studies have found different types of ncRNAs to be involved in OC. Among these ncRNAs, miRNAs have been used to predict the progression, relapse, and patient survival of epithelial OC by real-time quantitative PCR (RT-qPCR) [61,62]. Using small RNA-seq in 179 serum samples from epithelial OC patients, Elias et al. performed a neural network analysis to identify relevant circulating miRNAs for early OC detection [63]. They described seven miRNAs able to detect disease even with tiny tumors, suggesting that this method could be used to detect OC even in the smallest tumors. More recently, another study analyzed the involvement of lncRNAs in HG-SOC [64]. They identified four lncRNAs associated with patient survival and seven with response to chemotherapy, where four predicted a worse response and three predicted a good response. These studies indicate that ncRNAs play an important role in OC and could be used either for early detection of the disease or as predictive biomarkers of response to chemotherapy.

### 3.2. Unraveling OC Chemoresistance Using RNA-Seq

Several studies have used RNA-seq to shed light on the mechanisms of chemoresistance in OC or to overcome such resistance (Table 2). One study used patient-derived xenograft (PDX) models to study chemoresistance in OC to carboplatin/paclitaxel treatment [65]. PDX tumors were found to recapitulate the heterogeneity of the original tumors, and after treatment, they show a differential genetic profile that suggests that few but consistent pathways mediate chemoresistance, consistent with recent results [66]. Cohen et al. showed that IRF1, a transcription factor that works on immune system regulation, was associated with platinum sensitivity and improved patient survival, acting as a tumor suppressor gene and a predictor of patient survival [44]. Another study conducted a high-throughput combinational screen and RNA-seq in OC cells and identified dual oxidase maturation factor 1 (DUOXA1) as upregulated in platinum-resistant OC cells [45]. DUOXA1 overexpression led to increased reactive oxygen species (ROS) production and activation of the ATX-Chk1 pathway, and Chk1 inhibitors suppressed cisplatin resistance.

Chemoresistance in OC has also been related to particular miRNAs. In line with the latter study, the miRNA miR-137 was recently identified as being expressed at low levels in cisplatin-resistant OC cells [46]. In these cells, miR-137 expression is suppressed by c-Myc, which targets EZH2 mRNA, therefore leading to increased EZH2 expression. Inhibition of the c-Myc-miR-137-EZH2 axis suppresses cisplatin resistance, and the activation of this axis is sustained by ROS production mediated by DUOXA1. Wang et al. analyzed the function of miRNAs in the DNA damage response (DDR) in OC [67]. They generated a score based on 10 miRNAs that predicts not only defects in HR and genome instability but also a better response to platinum-based compounds, proposing an alternative method to identify patients with HR defects that are more likely to have a good response to platinum.

Other ncRNAs have been related to OC resistance. Song et al. analyzed the expression of lncRNAs across multiple patient databases with microarray and RNA-seq data [68]. They found a 7-lncRNA signature with high sensitivity and specificity that was able to predict patient survival independently of the HG-SOC molecular subtype. A recent study used Bru-seq, which maps nascent RNA transcripts using bromouridine tagging, in OC cells treated with the gp130 inhibitor SC144 [47]. This compound led to upregulation of hypoxia response genes and the hypoxia-inducible factor antisense HIF1A-AS2 and sensitized OC cells to Olaparib, carboplatin and cisplatin. Finally, RNA-seq has also been used to detect circular RNAs (circRNAs), which are RNA molecules derived from noncanonical backsplicing events of pre-mRNA transcripts and whose regulatory role and involvement in cancer is emerging. A study identified the circRNA circANKRD12, containing exons 2 and 8 of the ANKRD12 gene, as highly expressed in breast cancer and OC and related to the invasion and migration of cancer cells [69].

Finally, few studies have used transcriptomics in OC in relation to therapy in patients with HR deficiency. Recently, Arakelyan et al. identified a distinct transcriptomic profile of BRCA1 and BRCA2-mutated breast and ovarian tumors using RNA-seq data from the TCGA [70]. PARP inhibitor gene signatures were in or near the deregulated gene clusters of the transcriptomic landscapes. Another recent transcriptomic study identified CXCL11 as upregulated in ovarian tumors with HR deficiency and suggested this gene as a biomarker for anti-PD-L1 immunotherapy [71].

### 3.3. A Single-Cell Perspective for the Study of OC Transcriptomics

RNA-seq represents a powerful tool for the investigation of tumor molecular biology and therapy resistance. However, bulk RNA-seq generates results based on a cell population that is often heterogeneous, especially when analyzing tumors, and this intratumor heterogeneity implies that different transcriptional signatures may exist within the same tumor, thus compromising the results of therapy [72]. In OC, recent reports have shed light on the composition of tumor cells, their microenvironment, and their evolution during chemotherapy. Shih et al. identified sixteen cell populations in fourteen OC samples with different grades and origins [73]. The proportion of these populations changed from primary to metastatic sites from epithelial to leukocytes. Another study analyzed primary Fallopian tube benign samples and identified 10 epithelial subpopulations with distinct transcriptomic programs [74]. They reconstructed differentiation trajectories from secretory epithelial cells and identified PAX8, SOX17, and RUNX3 as potential drivers of this differentiation, defining an ‘early secretory’ population that is likely the origin of HG-SOC.

Other studies have characterized the microenvironment of ovarian tumors, focusing on their degree of infiltration by immune cells. Olalekan et al. analyzed metastatic OC samples from the omentum of six patients and found cancer, stromal and immune cell types [75]. Two groups of samples were identified according to their high or low level of T cell infiltration, and their molecular characterization suggested an antitumor response in the highly infiltrated tumors. A very recent report characterized 15 ovarian tumors with different levels of immune infiltration that differed in their cell type composition [76]. They found chemokine receptor-ligand interactions as a potential mechanism driving immune cell infiltration. Both reports have major implications for immunotherapy in OC. Finally, Nath et al. investigated HG-SOC tumor evolution during chemotherapy using scRNA-seq and WGS of malignant ascites and pleural effusions [77]. They defined several transcriptional signatures or archetypes and found one of them characterized by high metabolic activity and proliferation, while there were no consistent genomic alterations defining those states. Interestingly, this metabolic and proliferative archetype was progressively enriched as chemotherapy progressed, and resistance to different lines of treatments was acquired.

## 4. Epigenomics of OC

Gene expression is regulated by processes that do not alter the DNA sequence and that are included under the term epigenetics. Epigenomics, which refers to the epigenetics of the whole genome, comprises reversible modifications such as DNA methylation or histone modifications (e.g., acetylation, methylation) (Figure 1), as well as regulation by ncRNAs such as miRNAs. Tumors frequently use epigenetic alterations to proliferate and escape anticancer treatments, and therefore therapies targeting the epigenome are being increasingly tested [78]. In OC, epigenetic alterations, including DNA methylation and histone modifications, have been described in recent years as important contributors to tumorigenesis and chemoresistance [79] (Table 2).

### 4.1. Alterations of DNA Methylation in OC

DNA methylation at carbon 5 of cytosines (5-methylcytosine, 5mC) is an important player in the regulation of gene expression that typically occurs at CpG dinucleotides of gene promoters [80] (Figure 1). Promoter DNA methylation is associated with gene repression and is regulated by DNA methyltransferases (DNMTs), which mediate de novo or maintenance methylation, and ten-eleven translocation (TET) proteins, which convert 5mC to 5-hydroxymethylcytosine (5hmC). In OC, as well as other types of tumors, two main methylation patterns have been found: hypermethylation of the promoters of tumor suppressor genes and hypomethylation of repeated sequences [81].

A study analyzed global CpG island (CGI) methylation and mRNA expression using microarray-based technologies in OC cell lines treated with increasing concentrations of cisplatin [82]. They found a strong correlation between drug resistance and the number of hypermethylated CGIs, as well as reversion of chemoresistance using DNA methylation inhibitors. Moreover, they identified cell adhesion and tight junction pathways as repressed by hypermethylation and proliferative pathways such as PI3K/Akt and TGF-β as activated by hypermethylation. Another study performed an integrated genomic, epigenomic, and transcriptomic analysis of OC cell lines [37]. They identified 96 genes with differential promoter methylation between normal Fallopian tissue and OC cells, and the latter genes were highly correlated with TCGA tumor samples, indicating that OC cell lines retain epigenomic modifications present in patients.

Regarding DNA hypomethylation, a passive demethylation mechanism that may be associated with genome instability and poor prognosis has recently been described [83]. Global DNA hypomethylation in epithelial OC was linked to advanced disease and a proliferative gene expression signature. DNA hypomethylation occurred in blocks falling into late replicating regions, lamina-associated domains, PRC2 binding sites, and histone H3 lysine 27 trimethylation (H3K27me3), indicative of a repressive environment. In addition, DNMT genes were downregulated in tumors with global DNA hypomethylation, supporting the passive demethylation model. DNA hypomethylation of repetitive elements, including LINE-1 and Alu elements, has also been described in OC tissues compared with the ovarian surface epithelium and the FTE [84]. Moreover, hypomethylation of LINE-1 elements was recently found in OC precursor lesions, suggesting their early deregulation from the p53 signature to the STIC and their subsequent overexpression in carcinomas [85].

Finally, global loss of 5hmC levels has been associated with resistance to platinum-based therapy and worse patient survival [86]. Both overexpression of TET2 and treatment with the DNMT inhibitor 5-azacytidine, which in turn enhances TET gene expression, increase global 5hmC levels and restore platinum chemosensitivity, suggesting the use of DNMT inhibitors in ovarian tumors with low 5hmC levels. Another DNMT inhibitor, guadecitabine, was used in combination with carboplatin in OC patients, showing a higher sensitivity to chemotherapy than carboplatin alone and better patient survival [87]. Although drugs targeting DNA methylation represent a promising strategy to sensitize ovarian tumors to chemotherapy, the broad effect of these compounds represents a challenge that requires precise approaches to avoid undesirable effects. On the other hand, loss of methylation specifically at RAD51C promoter, whose methylation and subsequent silencing is related to HR defects, has been recently shown to cause resistance to PARP inhibitor treatment in HG-SOC PDX models [88].

### 4.2. Histone Modifications, Regulatory Elements and Transcription Factors in OC

Another layer of gene regulation is provided by cis-regulatory elements (CREs), which are DNA sequences harboring transcription factor (TF) binding sites that control the expression of target genes [89]. CREs are basically composed of promoters and enhancers that act at long distances over target promoters, likely by a looping mechanism. Histone modifications at these CREs define their activation status and accessibility, which is due to the binding of TFs. The most commonly used ‘omics’ approach to inspect either histone modifications or TF binding genome wide is chromatin immunoprecipitation coupled to sequencing (ChIP-seq), which employs specific antibodies to isolate and sequence the DNA associated with these proteins (Figure 1).

Bivalent chromatin domains marked by histone H3 lysine 4 trimethylation (H3K4me3) and H3K27me3 usually silence developmental genes in embryonic stem cells, keeping them primed for activation during differentiation [90]. In OC, this bivalent mark has been associated with the silencing of genes in malignant tissues, including those belonging to the PI3K and TGF-β pathways [48]. Interestingly, these genes were also silenced in cancer stem-like cells and in chemoresistant OC cell lines, suggesting that epigenetic silencing associated with bivalent chromatin marks influences tumorigenesis and chemoresistance in OC (Table 2). More recently, genes associated with bivalent histone marks were found to show increased promoter CpG methylation and decreased expression after chemotherapy [91], reinforcing the idea that epigenetic silencing is a mechanism for the acquisition of chemoresistance. Histone H3 lysine 79 methylation (H3K79me) has also been linked to platinum resistance in OC [49]. Thus, the TF C/EBPβ recruits the methyltransferase DOT1L, which methylates H3K79, to promote chromatin accessibility and gene expression in drug-resistance genes. Another study identified the overexpression of the histone methyltransferases EHMT1 and EHMT2, which catalyze demethylation of histone H3 lysine 9 (H3K9me2) (associated with gene repression), as being responsible for PARP inhibitor resistance in HG-SOC [92]. Finally, the E3 ubiquitin ligase RNF20 and its target histone H2B have been linked to the origin of OC in FTE [93]. The heterozygous loss of RNF20 is common in HG-SOC, and its knockdown in FTE cell lines reduces H2B monoubiquitination (H2Bub1) and enhances cell migration, promoting chromatin accessibility and upregulation of immune signaling pathways that cause the migration phenotype.

Enhancers in OC have been found to contain single nucleotide polymorphisms (SNPs) that disrupt TF binding and are associated with OC risk variants [94]. Other studies have focused on the contribution of alterations in enhancers to chemoresistance in OC. Shang et al. reported that superenhancers, which comprise clusters of enhancers located in close proximity that control the expression of important lineage-defining genes, are more activated in chemoresistant OC cells [50]. They found SOX9 to be an important gene controlled by these upregulated superenhancers that are important to promote chemoresistance. In another OC cellular model, Ma et al. observed a redistribution of superenhancers during the process of drug tolerance after cisplatin treatment that potentially controlled the most misexpressed genes upon that treatment [51]. They also identified ISL1 as an important regulator of superenhancer plasticity that is suppressed during treatment.

A major TF involved in OC is Pax8, which controls the development of the Müllerian duct, including the fallopian tubes [95]. NGS-based omics techniques have allowed the recent investigation of its role in OC. Thus, it has been reported using RNA-seq and ChIP-seq that Pax8 chromatin binding is rewired in HG-SOC cells versus FTE cells, with altered genes frequently located close to Pax8 binding sites [96]. These Pax8 binding sites coincide with TEAD binding sites, which are TFs mediating the effects of the Hippo signaling pathway effector YAP1, suggesting a connection between Pax8 and Hippo. Interestingly, we have recently shown that the Hippo pathway is involved in chemoresistance in OC [97]. Another study identified an enrichment of SNVs in OC within CREs marked by histone H3 lysine 27 acetylation (H3K27ac), a mark of active enhancers and promoters, containing binding motifs of TEAD4 and Pax8 [98], confirming the importance of the Pax8 network in OC development. Other TFs that have been involved in OC are Sox18 [56], FoxM1 [99], FoxL2 [100], ETS1 [101], and Znf703 [102].

## 5. Concluding Remarks

The development of NGS-based omics techniques has revolutionized the study of cancer molecular biology and chemoresistance. Genomic, transcriptomic, and epigenomic approaches allow the identification of the genomic landscape, gene expression, and chromatin-mediated regulation of multiple tumors, including OC. Although the common resistance to chemotherapy of ovarian tumors and the late detection of the disease are still challenges for future investigation, the major limitation of the new discoveries by NGS in OC is the translation to clinical practice. Further efforts must be made to test new stratifications of patients based on their genomic, transcriptomic, or epigenomic features in clinical trials. This would allow us in future to apply a personalized medicine strategy in which the best available treatment option is given to the patients based on the determination of the BRCA mutational status. In any case, these exceptional tools will be very useful for future research and are essential to understanding unknown aspects of OC biology.

## Figures and Tables

**Table 1:** Histological, clinical, and molecular features of ovarian cancer subtypes. OS, overall survival; GIN, genetic instability; HR, homologous recombination.

Histology	Epithelial Subtypes	Type	Clinical Features	Molecular Features	Stages at Detection	5-Year OS
Epithelial (90%)	High-grade serous (70–80%)	Type II	High grade, aggressive, bilateral, disseminated to omentum and peritoneum	GIN, ubiquitous mutations in TP53 and frequent in BRCA1/2, HR deficiency	Stage III–IV	43%
	Low-grade serous (<5%)	Type I	Low grade (except clear cell), indolent, unilateral, restricted to ovary at diagnosis	No GIN, mutations in KRAS, BRAF, ERBB2, PI3K, PTEN, ARID1A, β-catenin	Stage I–II	
	Endometroid (10–15%)				Stage I–II	82%
	Mucinous (3–4%)				Stage I–II	71%
	Clear cell (10%)				Stage I–II	66%
Sex cord stromal cell (2%)			Indolent		Stage I–II	88%
Germ cell tumors (3%)			Indolent		Stage I–II	94%
Others or unspecified (5%)						

## Data Availability

No new data were created or analyzed in this study. Data sharing is not applicable to this article.
